# The Causation of Aneurisms. II

**Published:** 1895-04-20

**Authors:** A. Pearce Gould

**Affiliations:** Senior Assistant Surgeon to the Middlesex Hospital


### The Causation of Aneurisms.—II.

By A. Pearce Gould, F.R.C.S., Senior Assistant Surgeon to the Middlesex Hospital.

The second great cause of aneurism is increase in the arterial blood-pressure. This may result from plethora, from increased cardiac contraction, or from increased resistance in the capillaries and arterioles, or from a combination of two or more of these factors.

The influence of plethora in the production of aneurism is best seen by noticing that this disease is exceedingly rare in the anæmic and cachectic, and there is no other single condition which is such an absolute protection against aneurism as anæmia. Plethora greatly aggravates the pain and the other local symptoms of an aneurism, and increases its rate of growth; and under the heading of treatment we shall have to give a prominent place to well-ordered measures to reduce the quantity of the blood. Plethora acts indirectly as well as more directly in the causation of aneurism, for it tends to increase the vigour and force of the heart's action, and if long continued it may set up chronic renal disease.

The over-action of the heart may be result of physical, mental, or emotional excitement; aneurism is accordingly more common in the hard-working populations of temperate regions than in the more lethargic peoples of the tropics, and in no country is the disease so frequent as in the United Kingdom. Among us it is found especially in men, and in men who follow laborious employments, and particularly in those who are exposed to sudden severe straining effort—soldiers sailors, navvies. Heavy work when uniform and not interrupted by violent effort is not nearly so potent a cause of this disease as sudden effort. The whole cardio-vascular system accommodates itself to continuous hard work, but a sudden severe strain puts it out of gear. In some cases frequent outbursts of violent passion may play a part in causing aneurism.

The raising of the blood-pressure by increased arterio-capillary resistance occurs as a chronic condition in chronic renal disease, and in gout, and as an acute condition in severe muscular effort. It is important to note that increased arterial tension is the great cause of atheroma of arteries, and thus leads to aneurism in a double way, for it both induces a diminution of the resisting power and elasticity of the arteries, and exposes them to greater distending force.

From all the foregoing it is easy to understand that aneurism is many times more common in men than in women, although, with the greatly altered conditions of the two sexes, the large substitution of machinery for manual labour on the one hand, and on the other the larger share taken by women in active pursuits of all kinds, the proportion which has hitherto been found to exist—thirteen men to one woman—will probably undergo some modification. Age exerts a very potent influence. The perfectly healthy arteries of the young are able to withstand even the great strain thrown upon them by athletes, while at the other extreme of life, although the arteries are commonly diseased, the heart is so enfeebled that it puts no severe strain upon the vessels, and the weakness of the general muscular system protects the old from undue effort. It is the robust, otherwise healthy, men of middle age who suffer from aneurism.

Alcoholic excess is an important cause of aneurism; on the one hand it leads to chronic endarteritis—atheroma—and on the other hand it induces increased arterial tension partly by increased cardiac contraction and partly, in some cases, by the arterio-capillary obstruction associated with granular contracted kidney. Incidentally it also exposes its subjects to injuries of all kinds, and sometimes to violent strains and efforts. There is one curious exception to the rule that aneurism is more common in the male sex, and it is found in carotid aneurism. No sufficient explanation of the greater frequency of this aneurism in women than in men has been given; but the fact is undoubted. A very rare form of the disease—dissecting aneurism—has also been met with more often in women than in men. When aneurism occurs in the young it arises either from syphilitic disease of the artery or from the softening of an artery due to the lodgment of an embolus. In the latter case the embolism may be multiple and the origin of more than one aneurism. The favourite localities of aneurism afford striking evidence of the influence of strain and high blood-pressure in its causation. The most frequent seat of all is the arch of the aorta, and especially its convex right border. Other favourite seats are at the origin of a branch from a trunk, opposite the flexure of a joint, and where an artery lies against the bone. Again, aneurism is more frequent on the arteries of the right upper limb than the left, and this is true even of the subclavian artery, and while innominate aneurism occurs on the right, the disease has never been found on the corresponding intrathoracic portion of the left subclavian trunk. In the lower limbs no such preference for the right arteries is shown, nor should we expect it, for while there is usually a great difference in the work done by the right and left arms there is little or none in that accomplished by the two legs. This equality of work and vascular strain is not infrequently strikingly shown by the simultaneous occurrence of an aneurism in each popliteal space. Symmetrical aneurisms are also met with in the vessels at the base of the brain, but this is not to be wondered at so much as the infrequency of such an occurrence, when we remember how large a part general systemic conditions play in their causation.

### THE LEPROSY FUND.

Prizes of fifty guineas each have been awarded to the following gentlemen: Dr. George Newman; Dr. Edward Ehlers (Copenhagen); Dr. S. P. Impey (Robbin Island); Dr. Ashburton Thompson and Dr. James Cantlie (Hong Kong), for essays on the history and treatment of leprosy, sent in response to an invitation from the National Leprosy Fund. The adjudicators were Sir Joseph Fayrer, Sir Guyer Hunter, and Mr. Hutchinson. The successful essays will in due course be printed and published at the expense of the Fund.

